# Bleaching-Associated Changes in the Microbiome of Large Benthic Foraminifera of the Great Barrier Reef, Australia

**DOI:** 10.3389/fmicb.2018.02404

**Published:** 2018-10-09

**Authors:** Martina Prazeres

**Affiliations:** Marine Biodiversity Group, Naturalis Biodiversity Center, Leiden, Netherlands

**Keywords:** bleaching, ocean warming, foraminifera, symbiosis, microbial community

## Abstract

Ocean warming is known to cause detrimental effects in coral reef fauna that rely on photo-symbiosis for survival. Microbial associations can facilitate the success of species across a range of environmental conditions, and play a role in the capacity of organisms to respond to climate change. In 2016, the Great Barrier Reef experienced its third mass bleaching event, with sea surface temperature rising to 1.3°C above long-term monthly summer averages. Here, I investigate the effects of ocean warming on the chlorophyll *a* (chl *a*) content and microbiome of the large benthic Foraminifera *Amphistegina radiata*. Samples were collected in January and April 2016, before and after the mass bleaching event. In total, 71 specimens were collected from two different depths (6- and 18-m) to investigate depth-dependant responses associated with changes in chl *a* and microbiome. Pigment analysis showed a significant reduction in chl *a* between time points in specimens collected at both depths. Reduction in pigmentation was accompanied by changes in the microbiome, and a significant interaction of depth and time was observed. Genus-level bacterial community associated with *A. radiata* was significantly different across depth and time. However, ocean warming affected populations at both depths to a similar extent, and resulted in change from a *Betaproteobacteria*-dominated assemblage in January to a more diverse bacterial community by April. Analysis of presence/absence and relative abundance of bacterial taxa revealed significant differences between time points at both depths analyzed. OTUs classified as *Firmicutes*, which were either absent, or present in very low relative abundances (<0.1%) across all sample groups in January, were identified in abundances as high as ∼20% in specimens collected from 18-m depth in April. Class-level shifts were observed in shallow-dwelling specimens, from high abundances of *Betaproteobacteria* to a high abundance and diversity of *Actinobacteria*. These results demonstrate the sensitivity of LBF to the effects of ocean warming, for which depth did not provide protection, and highlights the capacity of LBF to re-assemble bacterial communities after a disturbance. This study provides the first molecular-based demonstration of changes in foraminifera-associated bacterial assemblages during a bleaching event on a natural reef system.

## Introduction

Across the globe, coral reefs are increasingly negatively affected by deteriorating environmental conditions, largely driven by ocean warming (Pandolfi et al., 2011; Hughes et al., 2017), in combination with local impacts such as terrestrial runoff (Pandolfi et al., 2003). Over recent years, the trend of increasing sea surface temperatures (SST) has been intensified by severe El Niño events and unusually warmer summers (Berkelmans et al., 2004; Pandolfi et al., 2011; Normile, 2016; Ampou et al., 2017). These phenomena have increased in frequency globally, resulting in mass bleaching events, driven by host digestion and/or expulsion of their algal endosymbionts, identifiable by color loss (Glynn, 1984; Kleppel et al., 1989; Hoegh-Guldberg et al., 2007; Hughes et al., 2017). The bleaching phenomenon was first observed among reef-building corals (Glynn, 1984; Hoegh-Guldberg et al., 2007), and has since been documented among other reef-dwelling calcifying organisms including large benthic Foraminifera (LBF) (Hallock et al., 2006).

Shallow reef-dwelling calcifiers play an important role in the reef carbon cycle, and as ecosystem engineers. Understanding their response to warming is essential when evaluating the fate of coral reefs under current climate change scenarios (Titelboim et al., 2017). LBF are key elements of the reef substratum (Hallock et al., 2003; Hallock et al., 2006; Lee, 2006; Langer, 2008), and can represent ∼5% of the total carbonate budget on the reef (Langer, 2008; Doo et al., 2017). They are fundamental for reef accretion and maintenance of sand cays and beaches across the Indo-Pacific region (Perry et al., 2011), and are likely to be affected by ongoing ocean warming. Additionally, their unicellular nature makes them excellent model organisms to study responses of symbiont-bearing organisms to climatic changes (Lee, 2006). Recent advances in foraminifera research have demonstrated the capacity of LBF to respond to changes in environmental conditions, as well as the presence of phenotypic plasticity within certain local populations (Prazeres et al., 2016, 2017b). Moreover, some species can be extremely heat tolerant, and are able to photosynthesise and calcify at temperatures of up to 36°C (Schmidt et al., 2016a,b).

Large benthic Foraminifera show a diverse and flexible association with prokaryotic organisms (Prazeres et al., 2017a), but can also acquire new eukaryotic symbionts to suit their environment across broad geographical scales (Momigliano and Uthicke, 2013). Microbial communities play a central role in the ecological stability of coral reef environments (Knowlton and Rohwer, 2003). Bacterial associations can facilitate the success of species across a variety of environmental conditions, playing a fundamental role in the evolution and adaptive capacity of eukaryotic organisms (Moran, 2007; Gilbert et al., 2012). Factors such as nutritional status, stress response, and disease are linked to shifts in the taxonomic composition of microbiomes and their hosts (Ainsworth and Gates, 2016). Association with both eukaryotic and prokaryotic organisms are likely to influence the ecological success of important reef-building organisms, such as corals (Little et al., 2004; Hernandez-Agreda et al., 2016) and Foraminifera (Prazeres et al., 2017a), and help these organisms to respond to ongoing ocean warming.

Previous studies demonstrated that changes in environmental conditions can cause a shift in the bacterial and algal communities of organisms, resulting in a loss of some specific taxa and appearance of novel groups (Webster et al., 2013, 2016; Prazeres et al., 2017a). These shifts can be associated with a change in temperature regimes, as demonstrated previously under lab-controlled conditions (Webster et al., 2016). In 2016, the Great Barrier Reef (GBR) suffered its third mass bleaching event, with SST up to 1.3°C above long-term monthly summer averages (Great Barrier Reef Marine Park, 2016; Hughes et al., 2017). This event represented a unique opportunity to investigate the responses of organisms to a natural warming event, and its effects on the microbiome of reef populations. Here I investigated the effects of increased SST on the microbiome of a common species of symbiont-bearing LBF, *Amphistegina radiata*. Samples were collected before and after the 2015–2016 mass bleaching event on the GBR, at two different depths to investigate any depth-dependant responses associated with changes in the microbiome. Chlorophyll *a* concentration of specimens was also analyzed to assess if loss of algal symbionts/pigment correlates with the host’s bacterial microbiome composition.

## Materials and Methods

### Field Collection and Temperature Profile

**FIGURE 1:**
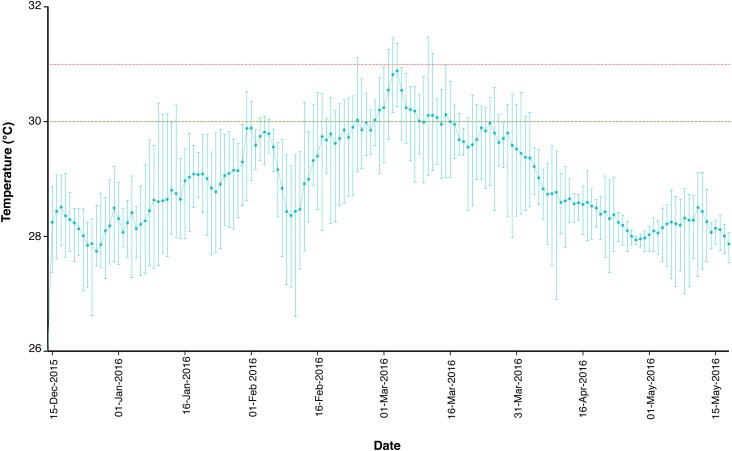
Temperature profile of the reef flat of Yonge Reef, on the GBR, Australia, between December 15, 2015 and May 15, 2016. Green and red dashed lines represent 30 and 31°C threshold, respectively. Data are expressed as mean, maximum and minimum daily temperature values. Data available at: http://data.aims.gov.au.

Specimens of the LBF *A. radiata* (Fitchel and Moll, 1798) were collected by SCUBA divers as previously described (Prazeres et al., 2016) at 6- and 18-m water depth (corrected to lowest astronomical tide levels), from Cod Hole (14° 39.9′ S; 145° 37.5′ E) in January and April 2016, and were taken before and after a major bleaching event on the GBR over the austral summer of 2015–2016 (Hughes et al., 2017). Cod Hole is located on the North end of Ribbon Reef #10, which sits ∼50 km offshore, exposed to little to no influence of terrestrial runoff (Brodie et al., 2007). The offshore region of the Northern section of the GBR experienced warming from December until mid-March, when temperatures began to cool (**Figure 1**). Surface water temperature values were obtained from December 2015 to April 2016, and were recorded at the reef flat of Yonge Reef, which is the closest reef from the collection site (ca. 10 km). Temperature data was recorded by the weather station maintained by the Australian Institute of Marine Science (Townsville, Australia), and freely available online^1^. In total, 23 specimens were collected in January (11 and 12 individuals at 6- and 18-m, respectively), and 48 in April (22 and 26 at 6- and 18-m, respectively). Samples were immediately placed in 4% paraformaldehyde (PFA) diluted in 3× phosphate buffer saline (PBS) solution at 4°C for preservation of the specimens. After ∼16 h, the 4% PFA solution was discarded and specimens were kept in cold 3× PBS until further processing. In the laboratory, specimens were washed in sterile 3× PBS, and placed in 90% acetone for chlorophyll *a* extraction without destruction of the specimen, which were then utilized for analysis of the bacterial microbiome.

### Chlorophyll *a* Analysis

Chlorophyll *a* (chl *a*) analysis was performed to assess whether the loss of eukaryotic photosynthetic symbionts is associated with changes in the bacterial community. Chl *a* analysis was carried out according to Prazeres et al. (2017b). Specimens were weighed and placed in 90% acetone overnight at 4°C for pigment extraction. Absorbance was analyzed using 96-well quartz microplates at 630, 663, and 750 nm, and chl *a* concentration was calculated using the equations of Hosono et al. (2012). Chl *a* concentration was further normalized by protein content, which was estimated based on a standard curve. The standard curve was built by plotting the wet weight and protein concentration of 52 specimens of *A. radiata* (**Supplementary Figure S1**). Final chl *a* concentration was given as ng chl *a* per μg total protein.

### Sample Preparation and Genomic DNA Extraction

After extraction in acetone, specimens were dried and placed in tubes filled with 200 μl of lysis buffer (QIAamp^®^ DNA Micro Kit, Qiagen) containing Proteinase K, and crushed using a micro-homogeniser. Samples were incubated overnight at 56°C, and genomic DNA extracted using a silica-membrane-based nucleic acid technique (QIAamp^®^ DNA Micro Kit, Qiagen, Germany). Genomic DNA was further purified using the OneStep^TM^ PCR Inhibitor Removal (Zymo Research, Germany). Extracted DNA concentration and purity were quantified using DropSense 96 (Trinean, United States). Only 41 out of 71 samples produced enough purified genomic DNA suitable for microbiome analysis. In total, DNA was recovered from 12 samples collected in April at each depth (6- and 18-m), and in January only nine samples were collected from 6-m and eight samples from 18-m.

### Library Preparation, Sequencing, and Sequence Analyses

Bacterial 16S *r*RNA samples were PCR amplified from the genomic DNA template, and sequenced using the Illumina MiSeq250 platform. DNA was amplified targeting the hypervariable region V3–V4 utilizing the 341F-805R primers pair (Klindworth et al., 2013) with added Illumina adapter overhang nucleotide sequences. Appropriate negative (fresh 18.2 Ω Milli-Q H_2_O) and positive (*Escherichia coli*) controls were also utilized throughout.

A three-step 35-cycle PCR using TaqMan^TM^ Environmental Master Mix 2.0 (ThermoFisher Scientific, United States) was used under the following conditions: 95°C for 10 min, followed by 35 cycles of 95°C for 15 s; 50°C for 30 s and 72°C for 40 s; after which a final elongation step at 72°C for 5 min was performed. Following this PCR, all amplicon products were purified using NucleoMag^®^ NGS Clean-up and Size Select (Macherey-Nagel GmbH & Co, Germany). PCR products of each sample from the first PCR were labeled with unique MiSeq Nextera XT labels. The second PCR was performed on a three-step 10-cycle program under the following conditions: initial denaturation at 95°C for 10 min, followed by 10 cycles of 95°C for 30 s, 55°C for 1 min, and 72°C for 30 s; a final extension at 72°C for 7 min was performed. PCR products were measured using the QIAxcel (Qiagen, Germany), and equimolar pooling was done using QIAgility (Qiagen, Germany). Libraries were sequenced utilizing Illumina MiSeq platform using the 2 × 250 bp paired-end protocol yielding paired-end reads that overlap almost completely. The primers used for amplification contain adapters for MiSeq sequencing and single-end barcodes allowing pooling and direct sequencing of PCR products. MiSeq sequencing was conducted by Macrogen (Amsterdam, Netherlands). The obtained fasta files containing all amplicon sequences were deposited to NCBI under the accession number SRP155388. The sequence data were processed using the statistical program R v3.4.3 (R Core Team, 2017), using the workflow described in detail by Callahan et al. (2016b). Briefly, forward and reverse sequences lacking adaptors and primer sequences were checked for quality, trimmed, and filtered to remove low-quality sequence reads. Quality score cut-off point was determined based on quality of both forward and reverse sequence reads, maintaining the recommended overlap for merging the sequences. The DADA2 method was utilized for filtering, de-replication, chimera identification and removal, and merging paired-end reads (Callahan et al., 2016a). This method constructs an amplicon sequence variant (ASV) table, which is an analog of the traditional Operational Taxonomic Unit (OTU). A total of 861 ASVs were identified, and after chimera removal a total of 384 ASVs were retained, from now on called OTUs. Sequences were then classified using the RDP classifier (Wang et al., 2007), aligned, and taxonomy assigned and defined at 97% similarity against the curated 16S SILVA database (Pruesse et al., 2007). Any sequences that were not assigned were filtered out of the dataset. Phylogenetic tree was constructed using the package *phangorn* (Schliep et al., 2017), which uses a neighbor-joining tree, and subsequently fits a GTR+G+I (Generalized time-reversible with Gamma rate variation) maximum likelihood tree.

### Data Analyses

Statistical analyses and graphical representation were performed in R v3.4.3 (R Core Team, 2017). Differences in chlorophyll *a* concentration were analyzed using a Two-way Analysis of Variance (ANOVA), using the function *aov* of package *car* (Fox and Weisberg, 2010). Data were log-transformed to meet homogeneity of variance and normality, which were tested using Levene’s and Shapiro-Wilk’s test, respectively. “Depth” and “Date” were employed as fixed factors.

Differences in bacterial taxa associated with *A. radiata* populations collected from differing depths and collection date were analyzed using the packages *phyloseq* (McMurdie and Holmes, 2013), *vegan* (Oksanen et al., 2017) and *microbiome* (Lahti et al., 2017). Graphic representation of the results was generated using the package *ggplot2* (Wickham, 2009). Prior to the analyses, abundance data were normalized to the total number of counts per sample as relative abundance, and only OTUs present in at least 0.1% averaged across all samples were retained. Total richness estimator Chao 1 was used to assess bacterial diversity among group samples (**Supplementary Figure S2**). Differences within and between each depth and time point (i.e., collection date) were analyzed through a Two-way Permutation Multivariate ANOVA (PERMANOVA) using unweighted and weighted-UniFrac resemblance matrices (Lozupone et al., 2007) to account for presence/absence, but also abundance of OTUs between samples. “Depth” and “Date” were employed as fixed factors. PERMANOVA outcomes were based on 9,999 permutations using Type I Sums of Squares, and permutation of residuals under an unrestricted model. PERMANOVA was performed using the function *adonis* of the *vegan* package. Permutation test for homogeneity of multivariate dispersions was confirmed for the fixed factors “Depth” and “Date” using the function *betadisper* of the package *vegan* to confirm that PERMANOVA results were not due to differences in group dispersions, but due to differences in bacterial community.

An unconstrained Principal Coordination Analysis (PCoA) was used as a visual representation of the compositional differences among bacterial community associated with *A. radiata* populations from different depths and dates, using the unweighted and weighted-UniFrac distance matrices. Distribution of bacterial OTUs was plotted against the same PCoA plot to visualize phylum-, class-, and genus-level taxa that potentially drove differences among samples. To identify the stable, consistent bacterial taxa present in *A. radiata* specimens collected from different depths and dates, core microbiomes were identified using the package *microbiome*. Core microbiome was defined as the OTUs that were present in at least 50% of samples.

## Results

### Differences in Chlorophyll *a* Content Between Depths and Months

**FIGURE 2:**
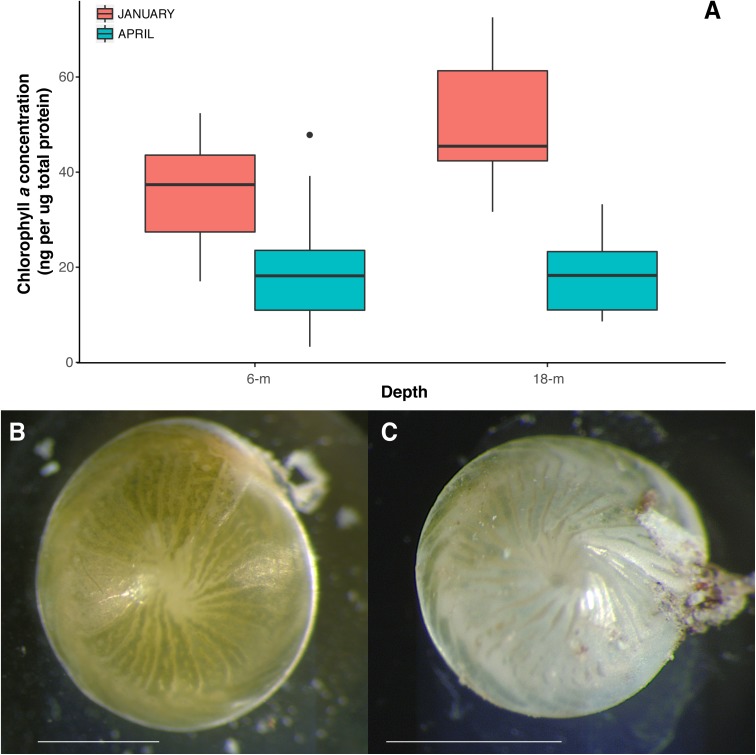
**(A)** Chlorophyll *a* concentration (ng per ug total protein) in *A. radiata* collected from at 6 and 18-m, in January and April 2016. Box plots represent median, the lower and upper quartiles (25 and 75%), while whiskers represent the minimum and maximum values. Images of specimens of *A. radiata* collected at 6-m depth in **(B)** January 2016 and **(C)** April 2016 showing signs of symbiont/pigment loss. Scales bar = 1 mm.

Results showed that specimens collected from 18-m water depth had significantly more chl *a* than those collected from 6-m (**Figure 2A**); pigment concentration was ∼50% higher at 18-m than 6-m in January. ANOVA results showed a significant effect of month of collection on the pigment concentration in *A. radiata* (**Table 1**), and a decrease in chl *a* concentration was observed at both 6- and 18-m specimens collected in January and April. Chl *a* concentration dropped sharply in *A. radiata* collected at 18-m, from 50.6 ± 3.7 (mean ± SEM) to 18.3 ± 1.53 ng chl *a* per μg total protein. Whereas concentration of photosynthetic pigment in specimens collected from 6-m sites showed a less pronounced reduction in chl *a* from 35.6 ± 3.3 to 20.3 ± 2.3 ng chl *a* per μg protein. The interaction between depth and date was also detected (**Table 1**). Changes in chl *a* concentration was also accompanied by visible external signs of symbiont/pigment loss (**Figures 2B,C**)

**Table 1:** Two-way analysis of variance results for chl *a* concentration of *A. radiata* populations collected at 6- and 18-m water depth, in January and April 2016.

Term	df	MS	F-ratio	*P*-value
Date	1	414	3.46	0.06
Depth	1	8790	73.40	**<0.001**
Date^∗^Depth	1	1127	9.41	**0.003**
Residuals	67	120		


*df, degrees of freedom; MS, means square; F-ratio, estimates of variance between and within samples. Significant values (*p* < 0.05) are in bold.*

### Bacterial Community Associated With *A. radiata*

**FIGURE 3:**
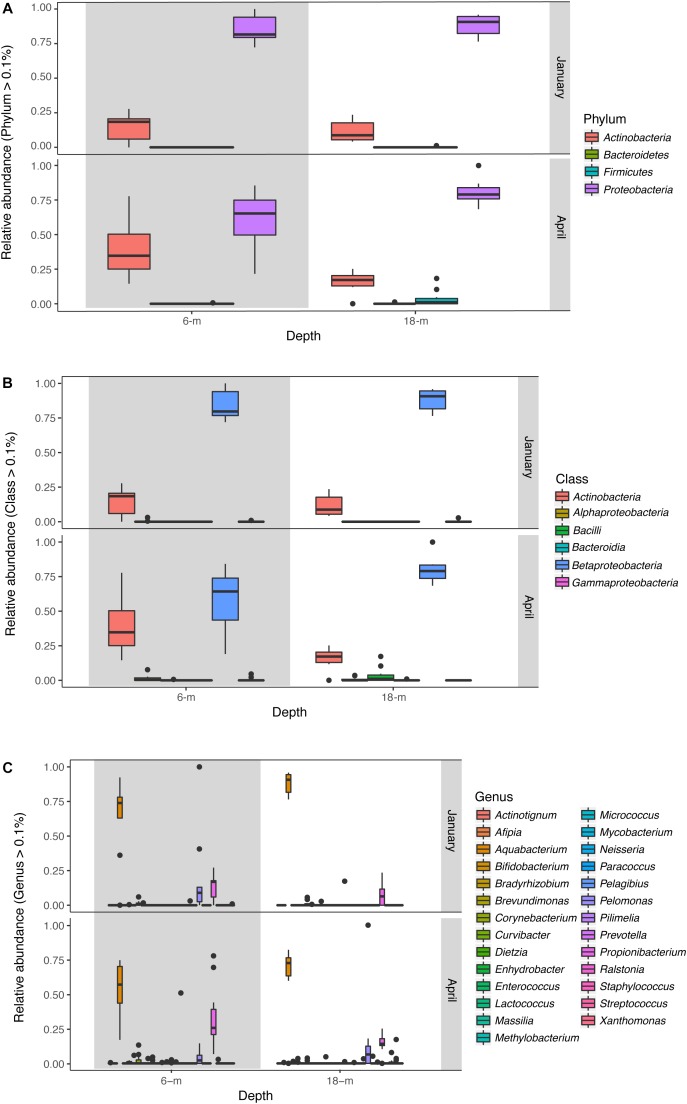
Relative abundance (OTUs >0.1%) of bacteria across samples collected at 6- and 18-m in January and April 2016 identified to **(A)** phylum, **(B)** class, and **(C)** genus levels. Box plots represent median, the lower and upper quartiles (25 and 75%), while the whiskers represent the minimum and maximum values.

**FIGURE 4:**
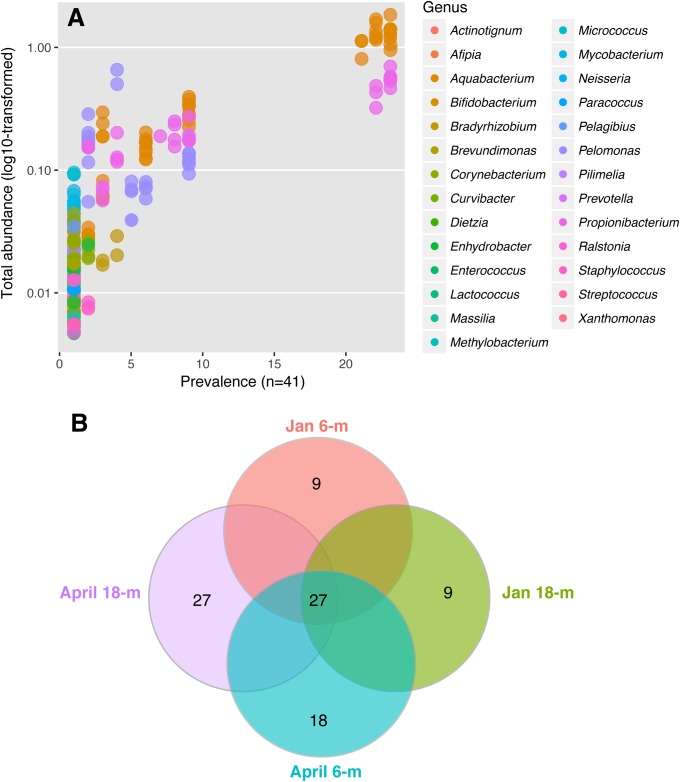
Bacterial community identified in *A. radiata* across different sample groups. **(A)** Prevalence of genera and total abundance of OTUs identified in the core microbiome across all individuals of *A. radiata*. Note that most OTUs are rare and therefore overlap, and the most common are represented by genera *Aquabacterium, Pelomonas*, and *Propionibacterium*. **(B)** Number of OTUs in the bacterial core microbiome (50%) across sample groups of specimens of *A. radiata* collected in January and April 2016 at 6- and 18-m during the mass bleaching event of summer 2015–2016 in the GBR, Australia.

The microbial community of *A. radiata* consisted of 384 identified OTUs. After the removal of singletons, and very low abundance OTUs (<0.1% averaged across all samples), a total of 260 OTUs remained across all samples, belonging mainly to the following bacterial phyla: *Proteobacteria* (75.97 ± 1.2%) and *Actinobacteria* (20.69 ± 1.3%) (**Figure 3A**). Among these OTUs, *Betaproteobacteria* were consistently the most abundant and diverse class of bacteria found across sample groups with an average relative abundance ranging between 69 and 73% (**Figure 3B**). Total number of OTUs identified per sample group did not differ greatly, ranging between 156 (January at 6-m) and 142 (January at 18-m) OTUs identified to genus level. Whereas in April, total number of OTUs was 145 and 148, at 6-m and 18-m, respectively (**Supplementary Table S1**). The bacterial genus *Pelomonas* (phylum *Proteobacteria*) that was absent in samples collected at 18-m in January, were found at a relative abundance of 12.1 ± 2.7% in April (**Figure 3C**). There was also a consistent increase in low abundance taxa at genus level between January and April observed in both populations (**Figure 3C**). Prevalence (i.e., number of samples in which a taxon appears) of OTUs did not reach values above 56% among samples and sample groups (**Figure 4A**). Core microbiome analysis revealed that no single OTU was shared among all samples (i.e., 100% coverage). In total, 27 OTUs were identified in at least 50% of the core microbiome (**Figure 4B**), and were classified as belonging to the genera *Aquabacterium* and *Propionibacterium*. Core microbiome composition and structure varied between sample groups, and an observed increase in rare bacterial taxa between January and April can be detected. For instance, samples collected in January possessed nine OTUs at each depth (6- and 18-m) identified in the core microbiome (**Figure 4B**). Samples from April showed a higher number of OTUs in the core microbiome at both 6- and 18-m samples (18 and 27 OTUs at 6- and 18-m water depth, respectively, **Figure 4B**). These OTUs were identified as mainly belonging to the class *Betaproteobacteria*.

**FIGURE 5:**
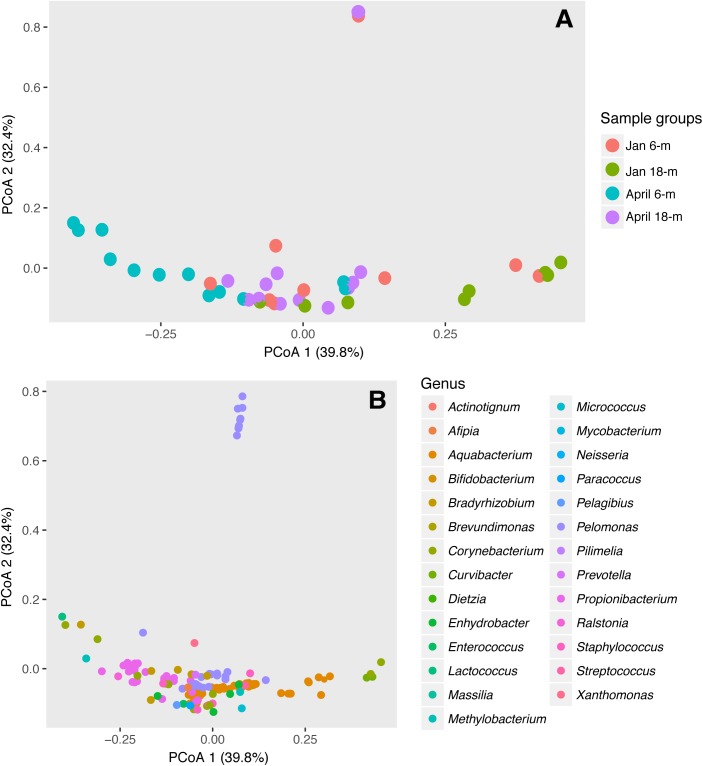
Differences in bacterial communities in *A. radiata* collect from 6- and 18-m water depth, in January and April 2016. Two-dimensional ordination plot utilizing weighted UniFrac-distance matrix showing the Principal Coordinate Analysis (PCoA) analysis representing **(A)** individual samples (*n* = 41) and **(B)** OTUs identified to genus level.

The composition and abundance of bacterial communities between specimens of *A. radiata* collected at 6- and 18-m depth was significantly different, and both depths were affected by increases in water temperatures. Analysis of homogeneity of group variance showed no significant difference between samples (Depth: *F*_39,1_ = 1.08, *P*-value 0.31; Date: *F*_39,1_ = 0.003, *P*-value 0.96). Based on *P*-values, PERMANOVA analysis using both unweighted (**Supplementary Figure S3** and **Supplementary Table S2**) and weighted (**Figure 5A**) UniFrac-distance matrices showed significant differences between sample groups, and the interaction between depth and date was also significant (**Table 2**). The presence of outliers in the data, and the overlap of the range of variation between sample groups (**Figure 5A**), could have contributed to a low R-squared. The presence of outliers could have been due to ectobiont contamination, as specimens are found associated with sediment, or the high concentration of chl *a* may suggest those were healthy specimens that did not suffer bleaching. Multi-dimensional scaling analysis using weighted UniFrac-distance matrix showed a divergent trend among sample groups. Most samples collected in April at 6-m showed a clear separation and, to a lesser extent, those collected at 18-m, whereas samples at both depths collected in January seem to be more homogeneous. The first two principal coordinates explained 72.2% of the observed variation (**Figure 5A**). Shifts in the relative abundances of *Aquabacterium* (phylum *Betaproteobacteria*), and presence of the *Actinobacteria* genus *Propionibacterium* were the most likely drivers of differences observed among groups, given the overlap of OTUs and samples in the same multi-dimensional space (**Figure 5B**).

**Table 2:** Two-way Permutation ANOVA results for weighed UniFrac-distance matrix of bacterial community associated with specimens of *A. radiata* collected at 6- and 18-m water depth, in January and April 2016.

Term	df	MS	F-ratio	R-squared	*P*-value
Depth	1	0.40	3.97	0.08	**0.004**
Date	1	0.73	7.24	0.14	**<0.001**
Depth^∗^Date	1	0.27	2.72	0.05	**0.03**
Residuals	37	0.10		0.73	
Total	40			1.00	


*df, degrees of freedom; MS, means square; F-ratio, estimates of variance between and within samples; R-squared, proportion of the variance that is explained by an independent variable (Depth, Date, or the interaction of factors). Significant values (*p* < 0.05) are in bold.*

## Discussion

Mass coral bleaching events are predicted to become increasingly common over time, as temperature continues to rise (Glynn, 1984; Hughes et al., 2017). While most studies focus on the effects of ocean warming on invertebrates, such as reef-building corals, many other organisms crucial to the health of the reef ecosystem will be negatively affected. Here I show changes in the bacterial microbiome of *A. radiata*, and a significant decline in photosynthetic pigment (i.e., bleaching), likely driven by a sharp increase in SST on the GBR during the summer of 2015–2016 (Bainbridge, 2017; Hughes et al., 2017). These results show that both the algal symbiont and associated bacterial communities were significantly affected by rises in SST, and how both eukaryotic and prokaryotic communities are interlinked when responding to changes in environmental conditions.

The morphological and physiological effects of increases in temperature have been extensively studied in LBF [reviewed in Doo et al. (2014)]. However, these studies have been limited almost exclusively to controlled laboratory settings. While these experiments have suggested that UV light rather than temperature is the main driver of pigment loss in LBF (Talge and Hallock, 2003), it is likely that temperature is a major contributor to the bleaching responses in field populations, as demonstrated previously for *A. lobifera* and *A. radiata* (Prazeres et al., 2016, 2017b; Schmidt et al., 2011). This is particularly apparent as individuals of *Amphistegina* and other LBF species are commonly found attached to rubble, and are able to regulate light through their phototaxic behavior to avoid excessive exposure (Fujita, 2004). In addition, field populations have been shown to possess different tolerance thresholds for increases in SST. For example, populations adapted to stable conditions of temperature and light are more sensitive to heat stress (Prazeres et al., 2016). These populations are not only sensitive to the single-effect, but also to the combined effects of light and temperature (Prazeres et al., 2017b). Depth is considered to provide some protection from the factors triggering bleaching, as light scatters and attenuates rapidly, and temperature regime is considered to be more stable at increasing depths (Bongaerts et al., 2010; Bridge et al., 2013). While this has been speculated for reef-building coral communities, the differences between shallow and deep populations of LBF have not yet been analyzed.

Results strongly suggest that the increase in SST, which led to widespread coral bleaching on the GBR in the summer of 2015–2016 (Hughes et al., 2017), also affected *A. radiata* populations. Average SST values reached over 30°C in mid-March 2016 (**Figure 1**), and was likely responsible for the onset of bleaching in *A. radiata* specimens, rather than changes in light availability, and subsequent changes in the bacterial microbiome. *In situ* light measurements taken from reef sites located in the Northern section of the GBR showed that while SST was highest in March, light levels actually decreased toward the end of summer (Bainbridge, 2017). Although other factors could have contributed to the differences observed here, the remarkable thermal anomaly detected in the GBR was the main factor likely driving these responses. Both shallow and deep-dwelling individuals showed a decrease in chl *a*, and this effect was actually more pronounced in deep specimens (**Figure 2A**). This difference is possible due to the fact that shallow-dwelling individuals have abundant light available for photosynthesis, and do not require as many algal symbionts/pigments as individuals in deeper waters. This leads to a naturally reduced concentration of chl *a* in shallower individuals. Nonetheless, a significant reduction in chl *a* was observed between January and April in *A. radiata* collected from both depth strata, indicating that depth (at least to 18-m) did not lessen the effects of the increased water temperature.

Along with declines in overall chl *a* concentration, clear spatial and temporal differences were detectable in the stability of host-bacteria interactions. Differences between the 6- and 18-m populations showed that local microhabitat conditions and the physiological state of the host likely influence the composition and diversity of bacterial communities associated with *A. radiata*. Results also showed that the initial bacterial microbiome between 6- and 18-m was different, and both populations were affected by increase in SST between January and April. Previous studies showed that the combined effect of ocean warming and acidification can significantly shift the bacterial functional composition in species of LBF (Webster et al., 2013, 2016). Additionally, the identity of algal symbiont also influences diversity of bacterial assemblages in LBF. For example, *Heterostegina depressa* (a diatom-bearing species of LBF) switches between different *Acidomicrobiales* associates at different temperature and *p*CO_2_ levels (Webster et al., 2016), whereas the dinoflagellate-bearing species *Marginopora vertebralis* showed shifts from *Alphaproteobacteria* to *Planctomycetes*, driven mainly by increases in temperature (Webster et al., 2016). Our results corroborate the notion that regardless of the type of algal symbiont, heat stress consistently affects the bacterial microbiome of species of LBF, and involves loss of specific bacterial OTUs and the appearance of novel microbial groups.

**FIGURE 6:**
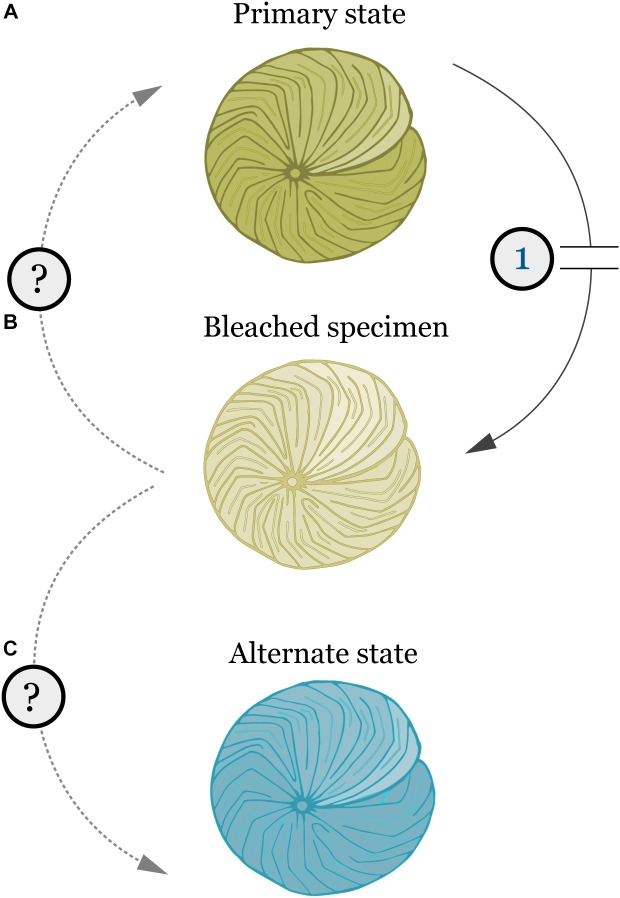
Schematic representation of the effects of bleaching and other physiological stresses (1) in the bacterial microbiome of *A. radiata* and other LBF. **(A)** Primary state (i.e., pre-stress) represents healthy specimens, with stable microbiome. **(B)** Bleaching leads to loss of algal symbionts, and shifts in the microbiome composition and OTU abundances. **(C)** Post-stress alternate state with different microbiome composition, and recovery of algal symbionts. Similar to the bacterial community, LBF might be able to acquire an alternate algal symbiont to suit their new environment.

Shifts in bacterial taxonomic groups were observed in specimens of *A. radiata* collected from 6- and 18-m, between January and April. Such changes in the composition of bacterial communities during bleaching periods may result in changes in the physiological function of these communities, and therefore affect host health, as observed for reef-building corals (Bourne et al., 2008). Following bleaching events, corals appear to actively select and shape their bacterial assemblages (Bourne et al., 2008; Ziegler et al., 2017). In the present study, the absolute number of individual OTUs among sample groups did not vary substantially. Post-disturbance these OTUs represented a wider array of genera, indicating an increase in bacterial diversity at the generic level. Local habitat has been reported to shape bacterial associations in field populations of LBF (Prazeres et al., 2017a). Therefore, it is possible that higher turnover of functional groups in the bacterial community of *A. radiata* post-disturbance can represent an opportunity for the host to acquire beneficial bacteria better suited to changed environmental conditions. It is also plausible that after the disturbance is removed, the bacterial assemblage may return to its pre-bleaching composition (**Figure 6**), as the host re-acquires bacteria that have been replaced.

Currently, the function of bacterial groups is still unclear. While a post-disturbance rise in bacterial diversity increases the likelihood of key functional groups appearing from which the host can select, it is still unclear whether or not this increase in generic diversity is a result of opportunistic parasitism, or just a relaxation of the selection by the host. It is possible that the physiological state of the host leads to an inability to develop or maintain a beneficial microbiome, which could result in the invasion of more opportunistic genera. Alternatively, the increase in genera could represent the clearing out of the existing community, and the subsequent re-colonization of a community more capable of assisting the host to respond to disturbances. Finally, LBF could also be utilizing bacteria as food sources (e.g., Bernhard et al., 2012). This group of benthic Foraminifera is mixotrophic, and depend on symbiosis with algae for energy intake utilized in growth and calcification (Hallock, 1985). Given the bleaching degree detected in April, it is plausible that these specimens could be relying on heterotrophic feeding through ingestion of bacteria to meet their energy requirements.

Analysis of core microbiome detected that no OTU was present across all samples, indicating a high level of individual flexibility of bacteria at all levels of analysis. However, a cut-off of 50% identified that at least 27 bacterial taxa are persistent, and present over time within and across some individuals collected from different populations of *A. radiata*. These results further support previous findings (Prazeres et al., 2017a), and suggest that a small proportion of abundant bacteria belonging to the same taxonomic unit can be found in most samples analyzed, but the majority of bacterial taxa occur at very low relative abundance, and are specific to a given population of *A. radiata*, which, in this case, are defined by the depth and time of collection. It is possible that high diversity of bacterial groups in the core microbiome is advantageous as ensures that functions restricted to certain taxa, such as nitrogen fixation or sulfate reduction, can continue to be performed when natural disturbances generate modifications of the bacterial assemblages (Shade and Handelsman, 2012).

In summary, these results show that shallow and deep populations of *A. radiata* display naturally different bacterial communities, unrelated to the temperature disturbance. However, depth was not a factor that protected *A. radiata* from the effects of changes in environmental conditions, and the loss of pigmentation and shifts in bacterial OTUs was consistent over both depths. The switch in bacterial composition, and the high level of low relative abundance OTUs post-disturbance reinforces the strong influence of algal symbionts (and consequently the bleaching response) on the microbiome composition across depth, which shifts from a stable bacterial community with lower number of OTUs to a more diverse assemblage. This phenomenon is indicative of a disturbance in the assemblage (Connell, 1978). However, it remains unclear if the bacterial microbiome will re-set to the primary state, or re-assemble in to an alternate state post-disturbance that would suit their physiological needs, and help them recover and cope with new environmental conditions (**Figure 6**). The high generic bacterial diversity revealed suggests that the LBF hosts are well positioned to be able to change their microbiome when conditions shift, and the bacterial microbiome is likely a key driver of ecological success for these crucial calcifying organisms on the GBR (Prazeres et al., 2017a). Identifying the factors that underpin the relationship between LBF and host-associated microbes is crucial, and will help to better understand the response of these organisms to ongoing climate change.

## Author Contributions

MP designed the experiments, conducted the laboratory analyses, analyzed the data, and wrote the manuscript.

## Conflict of Interest Statement

The author declares that the research was conducted in the absence of any commercial or financial relationships that could be construed as a potential conflict of interest.

## Footnotes

1 http://data.aims.gov.au
