# Supplementary material for: Bleaching-Associated Changes in the Microbiome of Large Benthic Foraminifera of the Great Barrier Reef, Australia
Source: Front Microbiol. 2018 Oct 9;9:2404. doi: 10.3389/fmicb.2018.02404 (PMC6189564; doi:10.3389/fmicb.2018.02404)
Supplement: Supplementary file 1 [file Data_Sheet_1.docx]

Bleaching-associated changes in the microbiome of large benthic Foraminifera of the Great Barrier Reef, Australia

Martina Prazeres

Marine Biodiversity Group, Naturalis Biodiversity Center, Leiden, the Netherlands

Email: [martina.prazeres@naturalis.nl](mailto:martina.prazeres@naturalis.nl)

Supplementary information


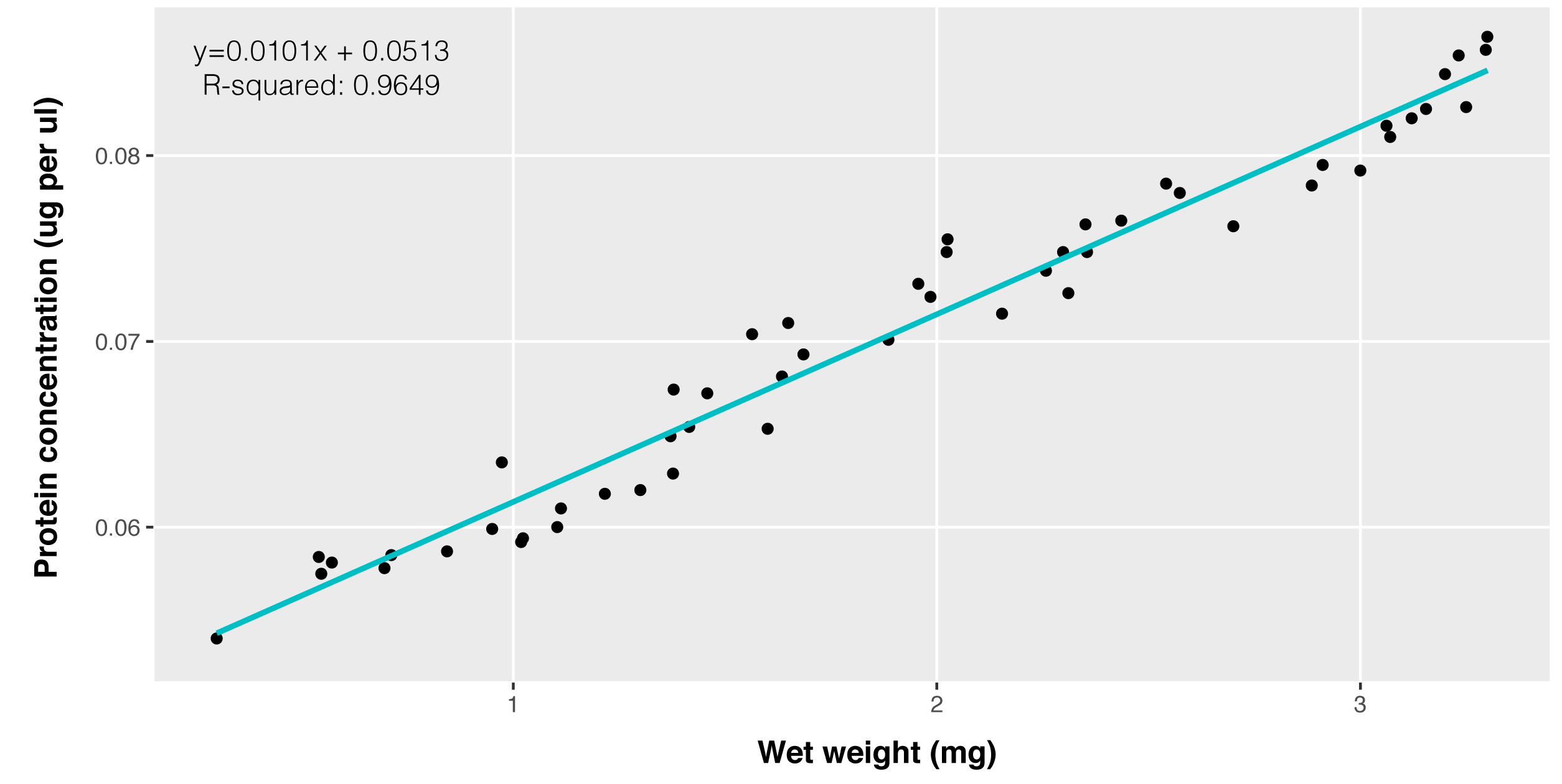


**Figure S1.** Scatter plot and linear regression line to estimate protein concentration (ng per ul) using wet weight (mg) as predictor variable. Correlation was performed using 52 specimens of *Amphistegina radiata*.


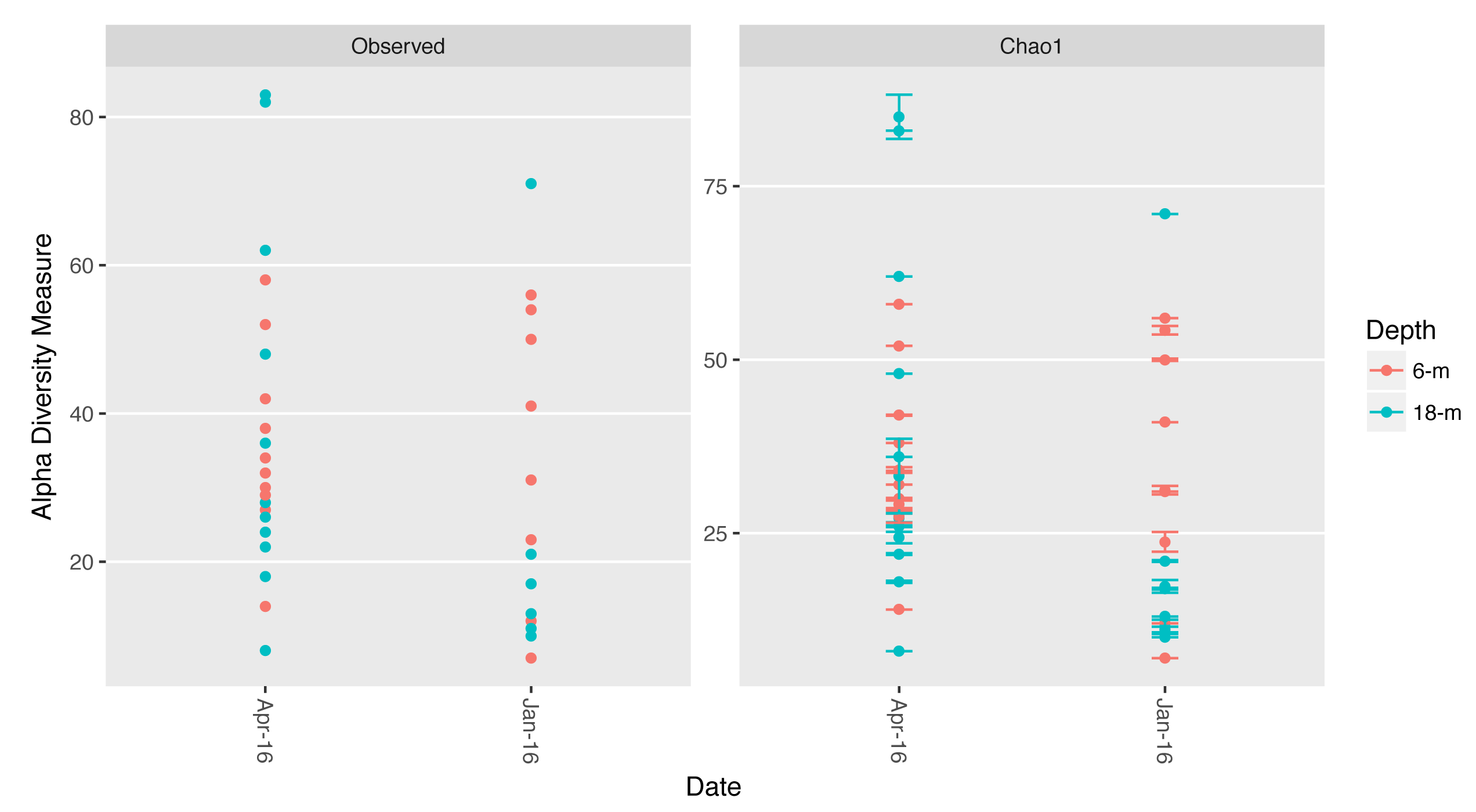


**Figure S2.** Species richness observed and estimated (Chao 1) of bacterial taxa associated with *A. radiata* among samples collected in January and April 2016, at 6- and 18-m.

**Table S1.** Total number of reads, and OTUs pre- and post-filtering per sample, removing singletons and OTUs that showed relative abundance < 0.1% averaged across all samples.

| **Sample ID** | **Depth** | **Date** | **Number of reads** | **Number of OTUs (pre-filtering)** | **Number of OTUs (post-filtering)** |
| --- | --- | --- | --- | --- | --- |
| Amphi 1 | 6-m | Jan-16 | 182963 | 30 | 30 |
| Amphi 2 | 6-m | Jan-16 | 248378 | 52 | 41 |
| Amphi 3 | 6-m | Jan-16 | 271759 | 32 | 32 |
| Amphi 4 | 6-m | Jan-16 | 193507 | 50 | 49 |
| Amphi 5 | 6-m | Jan-16 | 139983 | 26 | 26 |
| Amphi 6 | 6-m | Jan-16 | 215946 | 23 | 23 |
| Amphi 7 | 6-m | Jan-16 | 24519 | 11 | 11 |
| Amphi 8 | 6-m | Jan-16 | 157540 | 18 | 18 |
| Amphi 9 | 6-m | Jan-16 | 6311 | 7 | 7 |
| Amphi 10 | 18-m | Jan-16 | 531731 | 62 | 59 |
| Amphi 11 | 18-m | Jan-16 | 245593 | 83 | 47 |
| Amphi 12 | 18-m | Jan-16 | 344408 | 31 | 29 |
| Amphi 13 | 18-m | Jan-16 | 133153 | 10 | 10 |
| Amphi 14 | 18-m | Jan-16 | 232286 | 14 | 13 |
| Amphi 15 | 18-m | Jan-16 | 256198 | 22 | 21 |
| Amphi 16 | 18-m | Jan-16 | 319379 | 58 | 33 |
| Amphi 17 | 18-m | Jan-16 | 167776 | 29 | 29 |
| Amphi 18 | 6-m | Apr-16 | 221016 | 54 | 40 |
| Amphi 19 | 6-m | Apr-16 | 149210 | 27 | 27 |
| Amphi 20 | 6-m | Apr-16 | 352946 | 38 | 37 |
| Amphi 21 | 6-m | Apr-16 | 181316 | 42 | 42 |
| Amphi 22 | 6-m | Apr-16 | 218442 | 34 | 30 |
| Amphi 23 | 6-m | Apr-16 | 182064 | 17 | 15 |
| Amphi 24 | 6-m | Apr-16 | 90189 | 34 | 34 |
| Amphi 25 | 6-m | Apr-16 | 229939 | 24 | 12 |
| Amphi 26 | 6-m | Apr-16 | 5101 | 11 | 8 |
| Amphi 27 | 6-m | Apr-16 | 232368 | 28 | 28 |
| Amphi 28 | 6-m | Apr-16 | 190865 | 56 | 49 |
| Amphi 29 | 6-m | Apr-16 | 164024 | 28 | 27 |
| Amphi 30 | 18-m | Apr-16 | 162885 | 48 | 47 |
| Amphi 31 | 18-m | Apr-16 | 73503 | 12 | 12 |
| Amphi 32 | 18-m | Apr-16 | 217811 | 27 | 27 |
| Amphi 33 | 18-m | Apr-16 | 166430 | 71 | 60 |
| Amphi 34 | 18-m | Apr-16 | 283045 | 41 | 37 |
| Amphi 35 | 18-m | Apr-16 | 246961 | 36 | 36 |
| Amphi 36 | 18-m | Apr-16 | 45011 | 8 | 2 |
| Amphi 37 | 18-m | Apr-16 | 196519 | 17 | 14 |
| Amphi 38 | 18-m | Apr-16 | 246563 | 82 | 73 |
| Amphi 39 | 18-m | Apr-16 | 213512 | 13 | 13 |
| Amphi 40 | 18-m | Apr-16 | 57186 | 21 | 21 |
| Amphi 41 | 18-m | Apr-16 | 156108 | 31 | 27 |

**
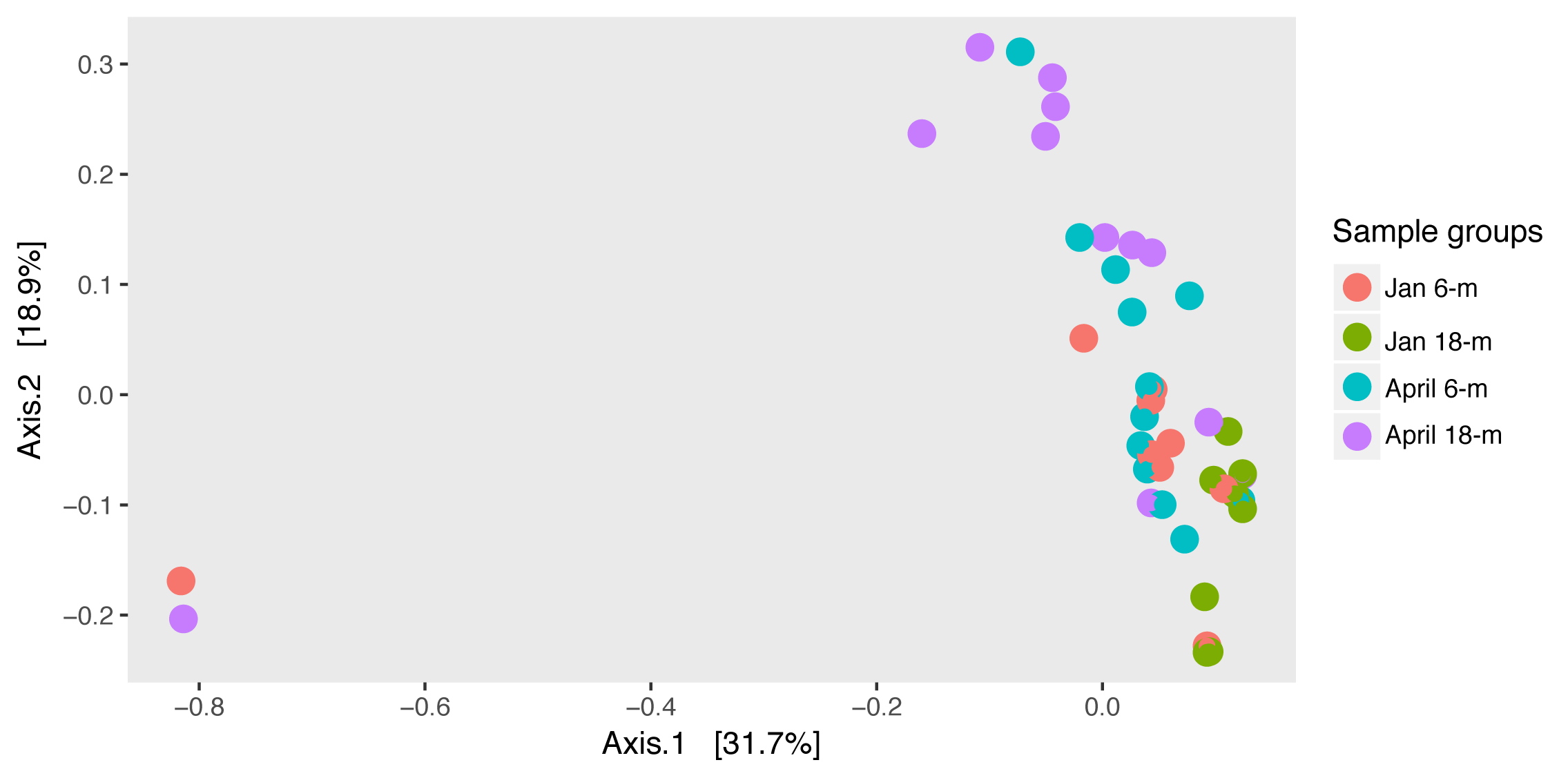
**

**Figure S3.** Principal Coordinate Analysis (PCoA) plot of unweighted-UniFrac distance matrix showing the first two principal coordinates that, combined, explain 50.6% of the observed variation.

**Table S2.** Two-way Permutation ANOVA results for unweighted UniFrac-distance matrices of bacterial community associated with specimens of *A. radiata* collected at 6- and 18-m water depth, in January and April 2016.

| **Term** | **df** | **MS** | **F-ratio** | **R-square** | **P-value** |
| --- | --- | --- | --- | --- | --- |
| Depth | 1 | 0.21 | 1.92 | 0.04 | **0.038** |
| Date | 1 | 0.32 | 2.87 | 0.07 | **0.003** |
| Depth*Date | 1 | 0.30 | 2.71 | 0.06 | **0.003** |
| Residuals | 37 | 0.11 |  | 0.83 |  |
| Total | 40 |  |  | 1.00 |  |
